# Genome Wide Analysis of Inbred Mouse Lines Identifies a Locus Containing *Ppar-γ* as Contributing to Enhanced Malaria Survival

**DOI:** 10.1371/journal.pone.0010903

**Published:** 2010-05-28

**Authors:** Selina E. R. Bopp, Vandana Ramachandran, Kerstin Henson, Angelina Luzader, Merle Lindstrom, Muriel Spooner, Brian M. Steffy, Oscar Suzuki, Chris Janse, Andrew P. Waters, Yingyao Zhou, Tim Wiltshire, Elizabeth A. Winzeler

**Affiliations:** 1 Department of Cell Biology, The Scripps Research Institute, La Jolla, California, United States of America; 2 Institute of Medical Biology, Singapore, Singapore; 3 The Genomics Institute of the Novartis Foundation, La Jolla, California, United States of America; 4 Department of Pharmacotherapy and Experimental Therapeutics, University of North Carolina School of Pharmacy, Chapel Hill, North Carolina, United States of America; 5 Leiden University Medical Center, Leiden, Netherlands; 6 Glasgow Biomedical Research Centre, University of Glasgow, Glasgow, United Kingdom; Queensland Institute of Medical Research, Australia

## Abstract

The genetic background of a patient determines in part if a person develops a mild form of malaria and recovers, or develops a severe form and dies. We have used a mouse model to detect genes involved in the resistance or susceptibility to *Plasmodium berghei* malaria infection. To this end we first characterized 32 different mouse strains infected with *P. berghei* and identified survival as the best trait to discriminate between the strains. We found a locus on chromosome 6 by linking the survival phenotypes of the mouse strains to their genetic variations using genome wide analyses such as haplotype associated mapping and the efficient mixed-model for association. This new locus involved in malaria resistance contains only two genes and confirms the importance of Ppar-γ in malaria infection.

## Introduction

Malaria infection by *Plasmodium falciparum* causes a variety of symptoms ranging from mild to severe. Previous studies suggest that the host genetic background plays an important role in susceptibility or resistance to severe malaria. Co-evolution of host and parasite has led to a wide variation of host-factors that influence the outcome of the infection. Alleles associated with sickle cell anemia, thalassemias, glucose-6-phosphate dehydrogenase deficiency, certain HLA haplotypes as well as allelic variants in the tumor necrosis factor cytokine and the CD36 scavenger receptor are all associated with resistance or susceptibility to malaria [1], [2], [3], [4], [5] and are found at higher frequencies in populations historically at risk for developing malaria.

In addition, several linkage studies using rodent malaria models related control of parasite levels in *P. chabaudi* infections to different malaria resistance quantitative trait loci (QTLs) (*char1-10*) on various chromosomes [6], [7], [8], [9], [10], [11], [12], [13]. Studies with *P. yoelii* confirmed the *char1* locus on chromosome 9 [14]. In addition, five loci have been associated with the development of experimental cerebral malaria (ECM) in *P. berghei* infections (berr1–5, cmsc and a locus on chromosome 18)[15], [16], [17], [18], [19] and one locus with malaria liver stage susceptibility (belr1) [20].

Traditional QTL analyses, typically an F_2_ cross, involving mice of two different parental origins are labor intensive and usually identify loci with dozens or hundreds of gene candidates. This is largely due to the limited genetic resolution of an F_2_ cross, unless large numbers of mice are used, and the fact that standard F_2_ crosses do not interrogate all of the available genetic and phenotypic variance in the mouse genome. On the other hand inbred mouse strains can be used to survey a wider array of phenotypic and genotypic differences. The inbred mouse strains are genetically identical within a strain largely as a result of breeding to homozygosity. As most are descended from relatively few progenitor lines they share haplotypes with one another. The haplotype structure of each of these strains has now been determined using dense sets of single nucleotide polymorphisms (SNPs) [21], [22]. Performing a phenotypic strain survey across inbred lines and then correlating phenotypic variations with the identified shared haplotype patterns or the SNPs directly, it is possible to map the genetic basis of a trait to a physical location in silico, in a process termed haplotype associated mapping (HAM) [23], [24] or efficient mixed-model for association (EMMA) mapping [25].

We used a lethal murine malaria model with *P. berghei* ANKA, a rodent malaria parasite. We infected mice from 32 different strains and measured various parameters such as body temperature, sequestration and survival. HAM and EMMA analysis were used to map the locus underlying survival after *P. berghei* infection in 32 different inbred mouse strains. The newly identified locus on chromosome 6 (*berghei* resistance locus 6, berr6) consists of only two genes and is thus much smaller than those identified with standard crosses.

There are very few complex traits that have been assayed in such a great number of strains and this is the first time a locus for malaria susceptibility has been identified with such a large-scale study. The strength of this analysis lies in the reduction of the normally large regions containing several hundred genes identified by conventional genetic analysis of a F_2_ cross to very narrow peaks with only a few possible gene candidates. In addition, we have generated a library of malaria-related phenotypes for uncharacterized mouse strains.

## Results

### Characterization of *P. berghei* ANKA infection in 32 different mouse strains

To identify genes involved in resistance or susceptibility to *P. berghei* infections we analyzed body temperature, schizont-load in organs, and survival in eight mice (four females and four males) from each of 32 inbred mouse strains. A list of the mouse strains with their group association and statistical analyses for all quantified phenotypes are summarized in Table S1.

#### Survival

**Figure 1:**
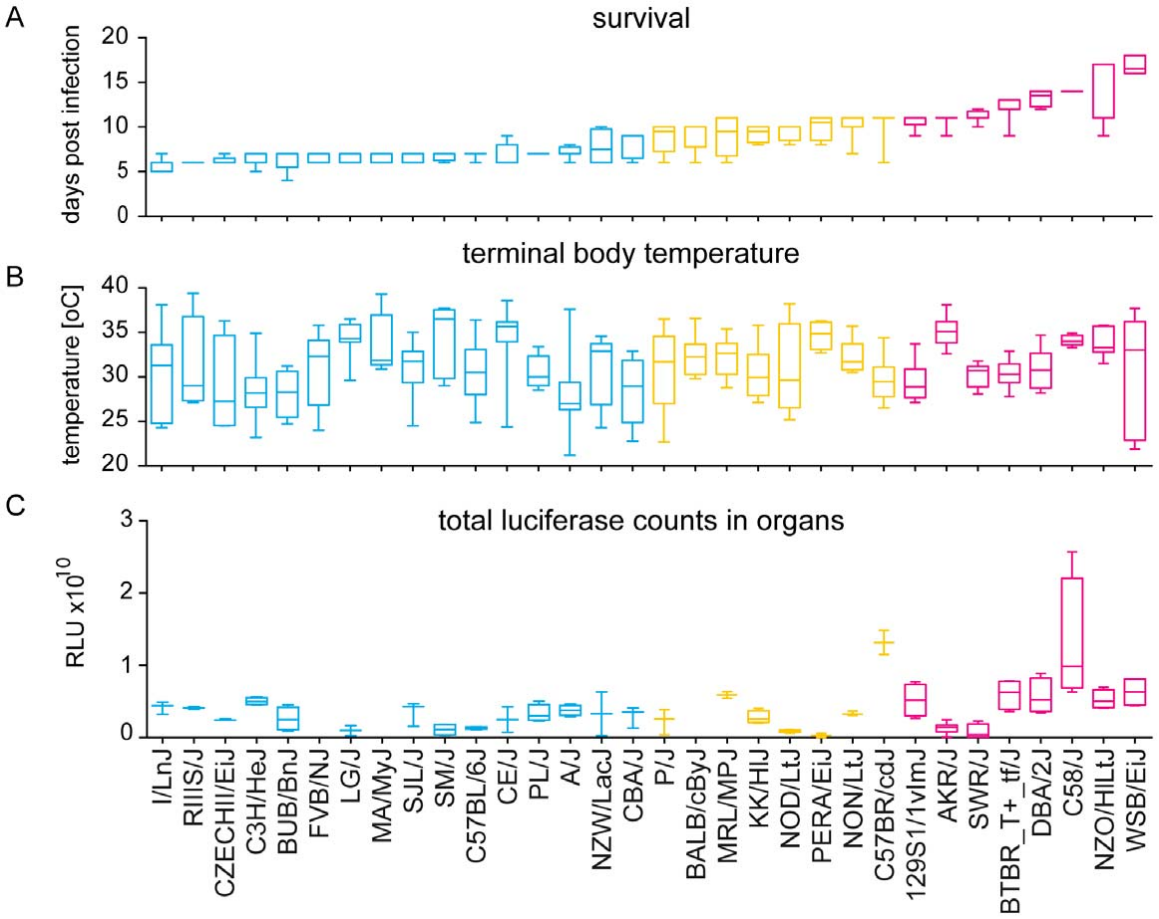
Summary of terminal disease phenotypes for *P. berghei* ANKA infection. Four males and four females from 32 different mouse strains were infected with *P. berghei* and survival (A) terminal body temperature (B) and total luciferase counts from dissected organs (C) were compared. Phenotypes are presented as box and whiskers plots with whiskers showing min to max. The name of the strain is given on the x-axis and the strains are listed for increasing average survival. The number of mice analyzed for each strain and the statistical relationship between different strains are shown in Table S1. The strains were grouped into three clusters according to their survival phenotype: susceptible (blue), intermediate (yellow) and resistant (red).

In our setup, the average survival of all mice was 9 (S.D. 3.1), however, 46% of all mice died before day seven. Mice that lived longer than seven days showed a normal distribution with a median of 11 days. Differences in survival between the 32 strains varied significantly. In order to simplify the analysis, we grouped the different strains into three clusters using a one-way ANOVA analysis followed by Tukey-Kramer posttest with alpha = 0.05. Two of these (susceptible and resistant) showed statistically significant differences (Figure 1A) and the remaining strains were placed in an intermediate cluster. The susceptible cluster (mean survival in the cluster 6.7) included I/LnJ, RIIIS/J, CZECHII/EiJ, C3H/HeJ, BUB/BnJ, FVB/NJ, LG/J, MA/MyJ, SJL/J, SM/J, C57BL/6J, CE/J, PL/J, A/J, NZW/LacJ and CBA/J. The resistant cluster (mean survival of the cluster 13.0) included 129S1/1vlmJ, AKR/J, SWR/J, BTBR_T+_tf/J, DBA/2J, C58/J, NZO/HILtJ and WSB/EiJ. The intermediate cluster (mean survival of the cluster 9.6) included P/J, BALB/cByJ, MRL/MPJ, KK/HIJ, NOD/LtJ, PERA/EiJ, NON/LtJ, and C57BR/cdJ). Three strains showed statistical differences between males and females from the same strain (Table S1).

To test if members of individual mouse strains grouped by ancestry, as shown in Table S1, are predominantly found in either the most susceptible, the intermediate or the most resistant cluster, the hypergeometric probability distribution for each group and cluster was calculated. Swiss mice and Castle's mice were significantly enriched in the most susceptible cluster (*p* = 1×10^−5^ and *p* = 3×10^−5^, respectively) while significantly excluded from the most resistant cluster (*p* = 0.002 and *p* = 0.004, respectively). The Japanese and New Zealand strains were significantly excluded from the most susceptible cluster and enriched in the intermediate survival cluster (*p* = 0.0001 and *p* = 0.0006, respectively). Bagg albino derivates mice were highly significantly excluded from the most resistant cluster (*p* = 7×10^−7^). Members of the three remaining mouse groups showed no preference for any cluster.

#### Body temperature

**Figure 2:**
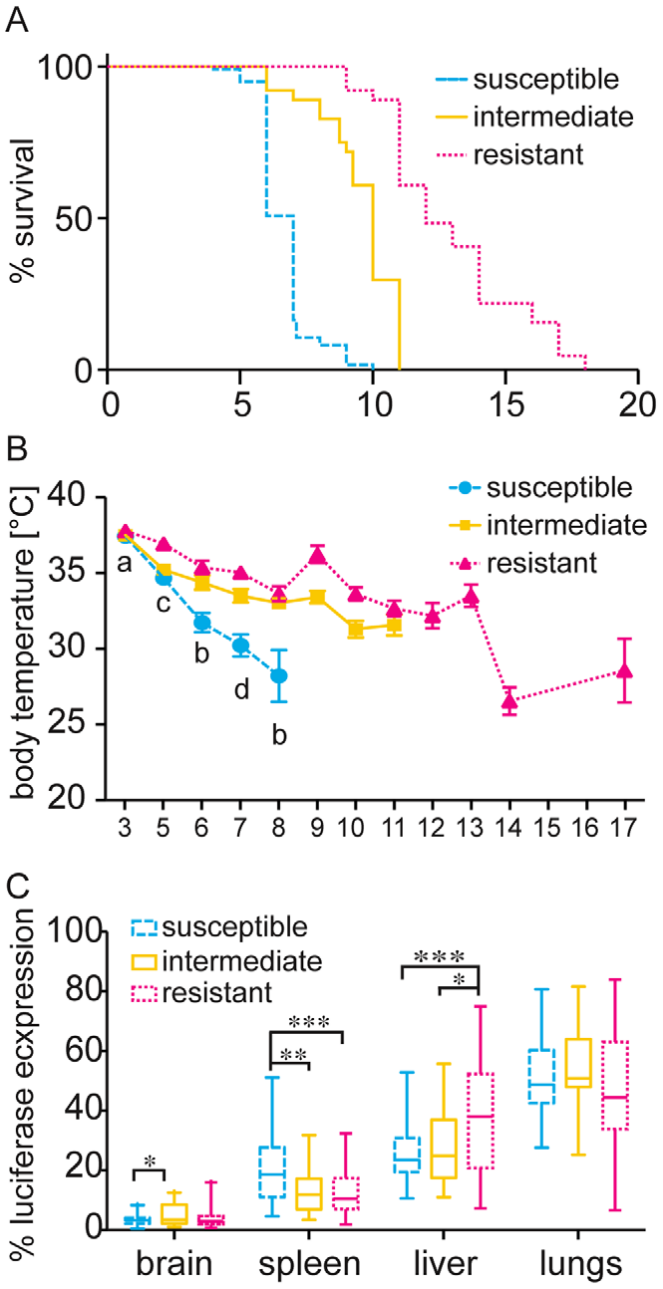
Differences between the susceptible, intermediate and resistant cluster. Survival and body temperature were recorded for mice infected with *P. berghei*. Mice were grouped into susceptible (blue dashed line, infected N = 122, dissected organs N = 42), intermediate (yellow solid line, infected N = 64, dissected N = 23) or resistant (red dotted line, infected N = 64, dissected N = 35) clusters according to their survival phenotype. A) Survival curve for the three clusters. Clusters were significantly different by a Log rank (Mantel Cox) test with p<0.0001. Average and STE for body temperature (B) over the course of infection with x-axis indicating time post infection. Statistical differences by one-way ANOVA followed by Tukey's posttest between clusters: a: susceptible versus resistant, b: susceptible versus resistant and intermediate, c: resistant versus susceptible and intermediate, and d: all three clusters differ. Luciferase expression of parasites in non-perfused organs was measured after dissection of organs from moribund mice. The luciferase expression per organ was expressed as the percentage of the total luciferase expression of all organs. C) Box and whisker plot with whiskers showing min to max relative luciferase expression for each organ. * indicates statistical significance of *p*<0.05 by one-way ANOVA analyses followed by Tukey's post test, ** indicates *p* between 0.01 and 0.001, *** *p*≤0.001.

In contrast to humans where a malaria infection is characterized by high fever, mice show a decrease in body temperature (hypothermia) upon infection with malaria parasites. Body temperature dropped significantly over the course of infection in all mouse strains (paired students t-test, *p*<0.04). With few exceptions, different mouse strains showed no significant differences in terminal body temperatures (for details see Figure 1B and Table S1). In four strains, female mice had significantly lower terminal body temperature than male mice from the same strain (unpaired students t-test *p*<0.03, for details see Table S1). In contrast there were significant differences in the course of hypothermia between the susceptible, intermediate and resistant clusters. Mice of the susceptible cluster had significantly lower body temperatures than mice from the resistant cluster from day three on (Figure 2B, one-way ANOVA followed by Tukey-Kramer post-test with alpha = 0.05). Over the course of infection the difference became more pronounced and mice of all three clusters showed significantly different body temperatures on day seven. Terminal body temperature of eight mouse strains correlated well with survival (for details see Table S1) but there was no correlation between terminal temperature and survival for all strains combined (R^2^ = 0.001).

#### Schizont distribution in organs

Sequestration of infected erythrocytes in the microvasculature of organs is associated with malaria pathology in humans and mice [26]. To test for differences in sequestration patterns between various mouse strains a transgenic *P. berghei* line (pbGFP-LUC_sch_) was used that expresses a GFP-luciferase fusion protein under the control of the schizont-specific promoter from the *ama*1 gene of *P. berghei* [27]. Because the invading merozoite imports some of the fluorescence into the erythrocyte, young rings also show some luciferase expression. Fluorescent imaging of the whole body of a living mouse or dissected organs detects therefore schizonts, the sequestering parasite forms, as well as early ring stages. Over the course of infection whole body luciferase counts in individual mice increased as expected (Figure S1). Expression of luciferase in brain, lungs, spleen and liver was analyzed in non-perfused, dissected organs from mice that were moribund. Only about 40% of the mice were sacrificed and the number of analyzed mice varied therefore for each strain and we did not analyze gender differences.

The sums of the luciferase counts of all organs from single mice were averaged for each strain and compared between strains (Figure 1c). Only C58/J and C57BR/cdJ had significantly higher total luciferase counts than any other strain (For details see Table S2). The resistant cluster had significantly higher luciferase counts than the susceptible one (one-way ANOVA followed by Tukey-Kramer post-test with alpha = 0.05). Total luciferase counts of the organs are an indicator of total parasite load in the host.

Photon counts from intact organs were also expressed as the percentage of the total photon counts of all organs per mouse (relative luminescence, Figure 2c). There were statistically significant differences in luciferase expression per organ for the different survival clusters (Figure 2C) while only a few individual strains showed significant differences from the other strains (Table S2). Significantly higher levels of luciferase expression in the spleen were associated with susceptible strains while significantly higher expression in the liver was associated with resistance. For statistical details of the individual strains see Table S2. Overall the luciferase expression was lowest in the brain, with intermediate mice displaying higher luciferase expression than susceptible mice, and highest in the lungs with no statistical differences between the clusters. In agreement with the findings above, there was a moderate negative correlation between survival and relative luciferase expression in the spleen and a positive correlation between survival and relative luciferase expression in the liver (R^2^ = 0.14 and R^2^ = 0.18, respectively). Correlation for relative luciferase expression in the remaining organs compared to survival, and all organs compared to terminal body temperature was low (R^2^>0.06).

In comparing the outcome of all the different phenotypic analyses, survival clearly showed the most robust discrimination between the different strains and pedigree groups (Figure 1). Therefore, survival was used as trait in the following analyses of the underlying genotype.

### Genome wide analyses identify a locus linked to host survival on chromosome 6

**Figure 3:**
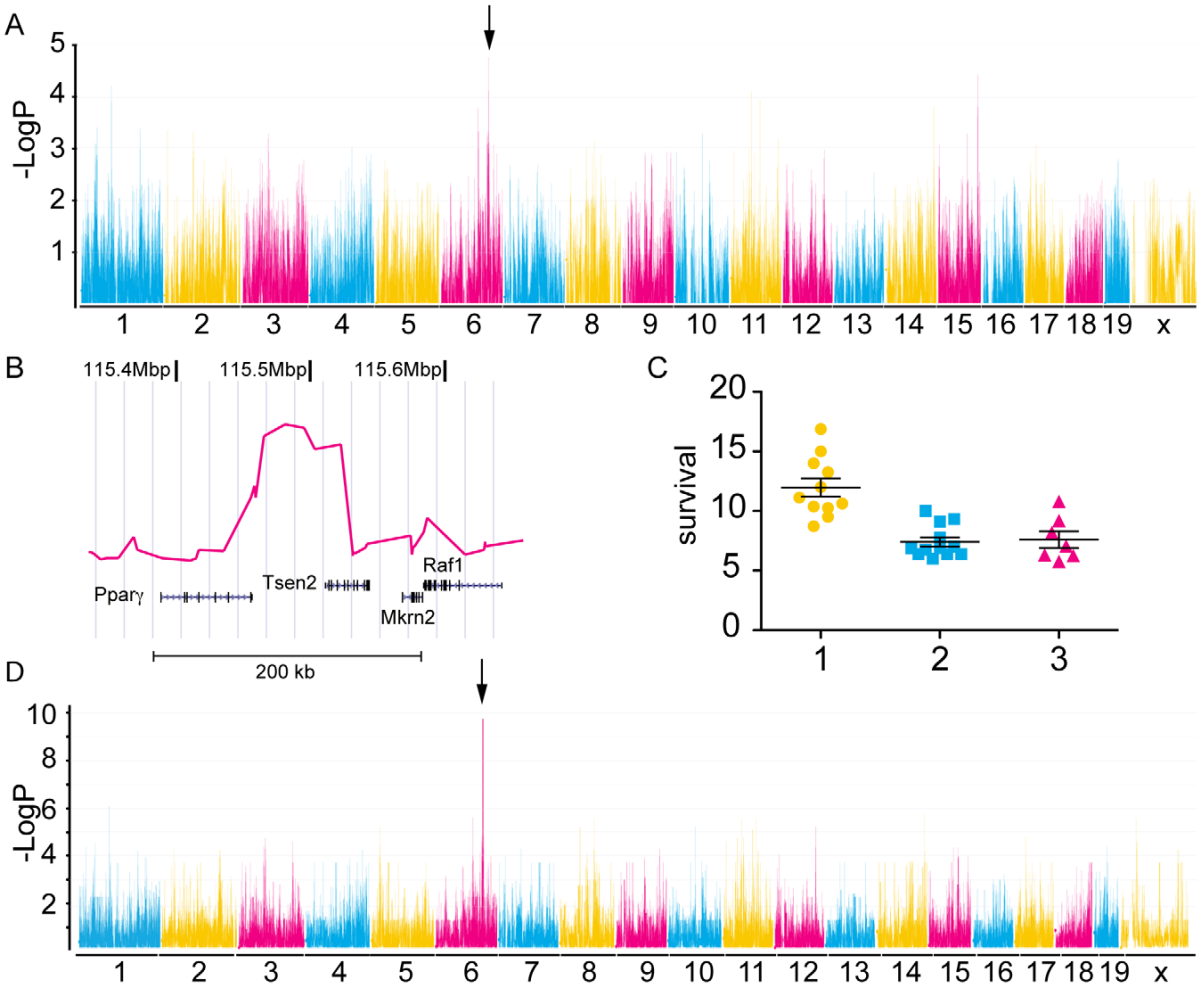
Genome wide analyses of average survival from 32 mouse strains. A) A genome wide scan with the haplotype associated mapping (HAM) algorithm using survival as trait of mice from 32 different mouse strains infected with *P. berghei* identified a locus with -Log_10_P score of 4.77 on chromosome 6 (arrow). The chromosomes numbers are indicated on the x-axis and -Log_10_P scores on the y-axis. B) Magnification of the locus on chromosome 6 identified by HAM analysis shows the –Log_10_P scores (red line) and the underlying genes and their chromosome positions. Three different haplotypes consisting of a combination of three SNPs were detected at the locus with the maximal –Log_10_P value of 4.77 (See also Figure 4a). The average survival of each strain carrying haplotype 1, 2 or 3 (yellow, blue and pink, respectively) are indicated in C). The efficient mixed-model for association (EMMA) method confirmed the locus on chromosome 6 identified with the HAM analysis (D). The –Log_10_P score of 9.87 with a false discovery rate of *q* = 3.5×10^−5^ is genome wide significant.

To link the survival phenotypes of the different inbred mouse strains to their genotype, two different genome wide analysis methods were used. The haplotype associated mapping (HAM) algorithm uses ANOVA to calculate the strength of genetic associations between an input phenotype and the ancestral haplotype structure (as inferred using a local window of three adjacent single nucleotide polymorphisms (SNPs) across the genome). A weighted bootstrap method is used to detect association peaks conditional on the population structure in the mouse diversity panel. At each genetic locus, the association score is represented as the negative log10-transformed P value. A score of –Log_10_P = 6 is a maximal score resultant from 10^6^ permutations performed at each locus. HAM analysis was performed for the survival phenotype across 32 strains using 297,674 informative SNPs. A locus with a –Log_10_P score of 4.77 was identified on chromosome 6 (115458884–115531474bp, NCBI M36) (Figure 3). Even though this –Log_10_P score is very good for this locus, it does not reach genome wide significance due to the conservative algorithm applied and the large number of SNPs tested. This locus contained the 3′ untranslated region (UTR) of peroxisome proliferator-activated receptor γ (*Ppar-γ,* GeneID: 19016, MGI: 97747) and the 5′ UTR of tRNA splicing endonuclease 2 homolog (*S. cerevisiae*) (*Tsen2,* GeneID: 381802, MGI: 2141599). In agreement with the nomenclature of previously identified QTLs [17], [18], we termed this newly identified locus on chromosome 6 *berghei* resistance loci 6 (berr6).

The locus identified with the HAM analysis was confirmed with the efficient mixed-model for association (EMMA) mapping model [25]. This modeling method uses a different algorithm for association of phenotype to genotype and essentially uses a single SNP association rather than an inferred haplotype structure. It also corrects for confounding factors like genetic relatedness and population structures by estimating the pair-wise relatedness between all individuals and fitting these to the vector of the phenotype thereby decreasing false positives and false negatives. The locus identified through EMMA at position 115494951 (–log_10_ *P* = 9.87), overlapped with the locus identified with the HAM analysis. This *p* value showed genome wide significance with a *q* value of 3.5×10^−5^. The q value is an estimation of the false discovery rate and was calculated as previously described [28]. For the mouse strains used in our study, this locus accounts for 53% of the variance in survival observed in females and 62% in males. While we tested other phenotypes as well, only body temperature on day 5 of male mice reached genome wide significance with a peak on chromosome 4 (123836655bp, -Log_10_P = 12.39).

While not much in known of the function of Tsen2, Ppar-γ forms a nuclear receptor heterodimer together with γ–retinoic X receptor (reviewed in [29]). It regulates transcription of a number of genes involved in adipose differentiation and metabolism, insulin sensitivity, bodyweight regulation, atherosclerosis and inflammation (reviewed in [30]). Ppar-γ has already been linked to malaria infection. An agonist of Ppar-γ enhanced the clearance of iRBCs of *P. falciparum in vitro* and improved the survival rate of mice infected with *P. berghei* and reduced parasitemia in the *P. chabaudi chabaudi* model in a CD36 dependent manner [31].

**Figure 4:**
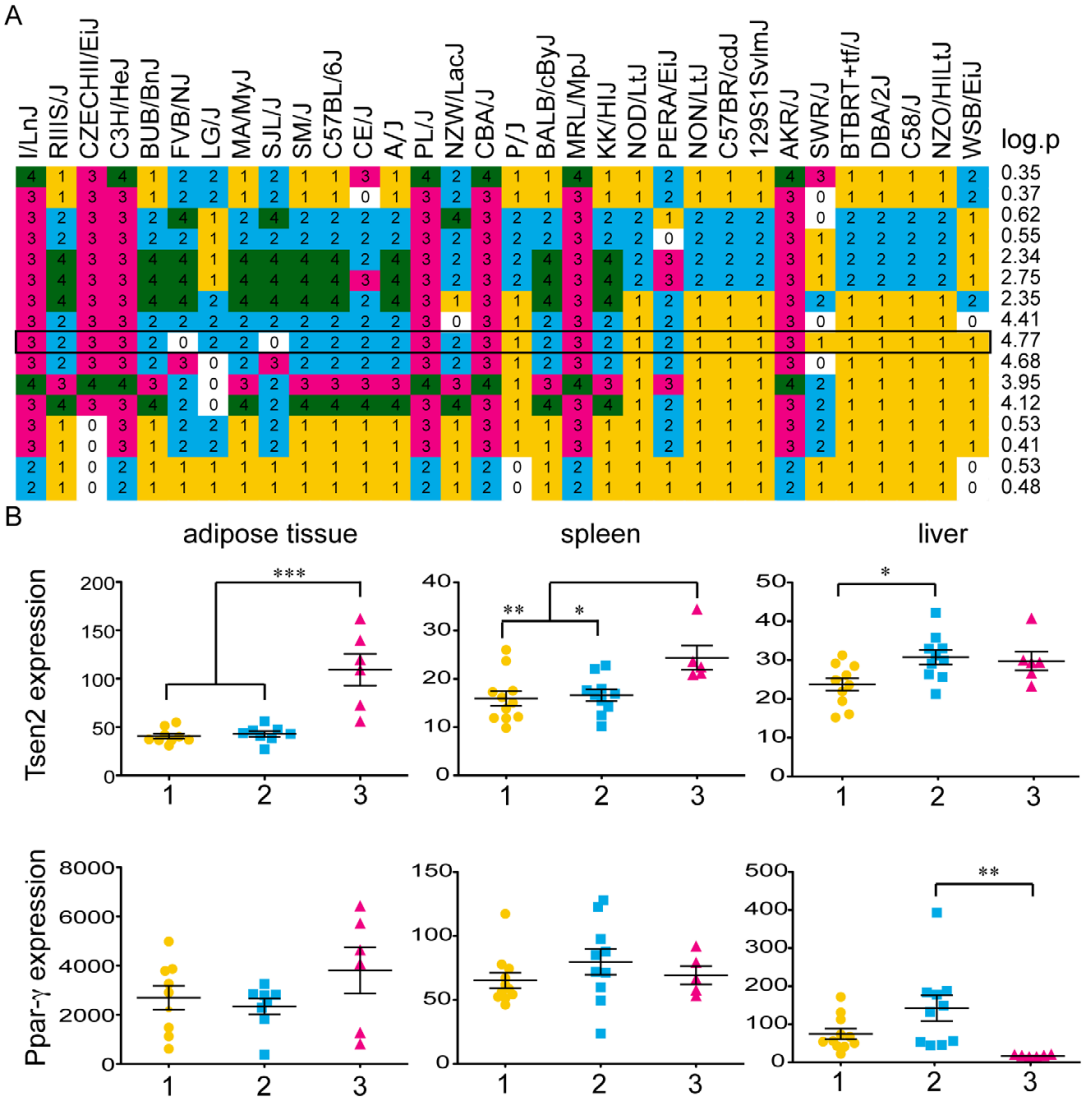
Haplotype distribution at berr6 locus and expression analysis of *Ppar-γ* and *Tsen2* haplotypes. The HAM analysis is based on the formation of haplotypes consisting of three consecutive SNPs. The compositions of haplotypes at different positions at the berr6 locus are indicated for each mouse strain (A). Each haplotype is indicated by a number and labeled in a different color. The mouse strains are listed according to their survival. –Log_10_P values for each position are indicated and the haplotypes with the highest score are framed. B) Expression patterns of *Tsen2* (upper panel) and *Ppar-γ* (lower panel) from different mouse strains were compared in adipose tissue, spleen and liver. The relative expression levels (y-axis) for each mouse strain were grouped and colored according to their haplotype at the locus with the highest –Log_10_P value (A). Significantly different expression levels were detected in all tissues for *Tsen2* but only in the liver for *Ppar-γ*. Scatter dot plots with the mean and the standard error for the expression of *Tsen2* and *Ppar-γ* haplotypes. * indicates statistical significance of *p*<0.05 by one-way ANOVA analyses followed by Tukey's post test, ** indicates *p* between 0.01 and 0.001, *** *p*≤0.001.

We compared the available expression data for uninfected mouse strains from the BioGPS [32], [33] portal of *Tsen2* and *Ppar-γ* from the spleen, liver, adipose tissue and hypothalamus with the haplotypes of the various mouse strains at the berr6 locus. *Tsen2* was significantly higher expressed in adipose tissue and the spleen of mouse strains sharing haplotype three while haplotype one showed significantly lower expression than two in the liver. *Ppar-γ* showed only significant expression differences in the liver where haplotype three had significantly lower expression than haplotype two (Figure 4). There were no differences in the expression levels in the hypothalamus (data not shown).

## Discussion

Genetic analysis of human populations can be difficult due to variations in haplotype structures between different ethnic groups, which can lead to false positive associations. The study of malaria in humans is even more difficult to conduct, as analysis of brain tissue or gene expression patterns in various organs is only possible in terminal cases. The mouse model has been shown to be a useful alternative to identify genes related to malaria infection in human populations. For example the protective aspect of pyruvate kinase deficiency was first identified in the mouse model [34] and has subsequently been linked to resistance in human populations as well [35]. The mouse model also allows testing the function and importance of identified candidate genes by using inhibitors, knockouts or allelic replacements.

Finding the best trait to distinguish different mouse strains is a prerequisite for the analysis of genetic association studies. We therefore first analyzed different phenotypes associated with infection of *P. berghei* ANKA in 32 different inbred mouse strains which have been bred to near homozygosity at almost all loci [36]. Hypothermia with temperature below 30°C is used as an indicator of experimental cerebral malaria (ECM) as it is associated with brain hemorrhage and early death in ECM susceptible mice [37]. A drop in body temperature towards the end of infection was however detected in all mice, indicating that hypothermia is not only associated with ECM but that it is a general marker for moribund mice in infectious disease models [38]. The body temperature on day 4 to 6 of infection was therefore better suited than the terminal values to predict survival. Therefore, the timing of hypothermia rather than the presence itself is predictive for disease severity.

The sum of the luciferase activity in various organs was significantly higher in the resistant cluster than in the susceptible one. This probably reflects generally higher parasitemia in the resistant cluster due to the longer persistence of the parasite in the host. Nevertheless, by comparing relative luciferase activity in various organs we found that susceptible mice had significantly more parasites sequestering in the spleen than the other two groups. The spleen plays an important role in malaria infection and it has been shown that splenectomized mice infected with *P. berghei* K173 survive longer than non-splenectomized mice in a strain specific manner [39]. Macrophages and dendritic cells presumably prime CD4^+^ and CD8^+^ T cell in the spleen and depletion of CD4^+^ and CD8^+^ T cells protect mice from ECM [40]. The accumulation of parasites in the spleen of susceptible mice might therefore prompt a stronger activation of the immune system due to a greater number of parasites being presented to T cells. This in return could result in tissue damage in various organs leading to early death. In contrast to the spleen, antigen presentation in the liver induces tolerance, known as portal vein tolerance [41]. Indeed, we observed that resistant mice had significantly higher luciferase expression in the liver than susceptible mice. Mice of the intermediate cluster had significantly more parasites in the brain than susceptible ones. Sequestration of parasites in the brain is mainly associated with human cerebral malaria (CM) but has also been reported in mice [42], [43]. As we do not have data on cytokine levels, lymphocyte counts or any ECM markers for the different mouse strain, death could be due to various reasons. However, the location of sequestration and its ability to induce an immune response might play a role in the severity of the disease and early death. The mechanism underlying the different sequestration pattern needs to be determined.

Survival was the only phenotype that showed low variability within a strain and significant differences between strains. The importance of the influence of the genetic background on survival was strengthened by the fact that members of closely related groups shared the same survival phenotype. Due to the lack of data on pathology we do not know which mice died of ECM, renal failure, pulmonary edema, anemia or any other complication associated with malaria [44].

Mice that died early showed low total luciferase counts in organs suggesting low parasitemia. These mice showed also an early drop in body temperature. These are signs normally associated with ECM but in the absence of further evaluation we cannot be certain. Resistant mice died with higher total luciferase counts suggesting high parasitemia. Body temperature dropped in resistant mice towards the end of infection. It is tempting to speculate that these mice did not die due to ECM but rather hyper-parasitemia. The intermediate cluster shows a similar course of body temperature as the resistant mice but their total luciferase counts are more similar to the susceptible mice. It is possible that mice of the intermediate cluster died due to complications other than ECM or hyper-parasitemia. In addition we found different organ specific relative luciferase counts between the three clusters suggesting that the location of sequestration might influence the outcome of infection. Some strains showed interesting divergence from their survival cluster indicating possibly new phenotypes. AKR/J mice of the resistant cluster survived on average 11 days, had low total luciferase counts, and a small drop in body temperature.

Even though different mouse strains might die due to different pathological reasons, we mapped survival to a new locus on chromosome 6, (berr6) between *Ppar-γ* and *Tsen2* using genome wide analysis such as HAM and EMMA. While the peak was detected with both analysis methods, it reached genome wide significance only with the EMMA method. The HAM analysis uses a more conservative approach and does not take the genetic relatedness of the strains into account. The haplotype conferring resistance is shared between unrelated mouse groups whereas the more closely related Swiss mice and Castle's mice predominantly share the susceptible haplotypes. *Ppar-γ* expression is differentially regulated in various tissues with highest expression in adipose tissue and it regulates expression of many genes including CD36. The innate immune response plays a major role in the clearance of infected erythrocytes and controlling progression to fatal disease [45]. Signaling through pattern-recognition receptors such as Toll-like receptor 2 (TLR2) and TLR9 and scavenger receptor CD36 on macrophages induce the release of proinflammatory cytokines. On the observation that macrophage CD36 helped control replication of blood stage parasites and enhanced survival of the host, Kain and his group hypothesized that “pharmacological modulation of innate immunity through pathways involving CD36 and related pattern recognition receptors might increase parasite clearance, modify deleterious host inflammatory responses to infection, and improve outcome” [31]. As CD36 transcription is regulated by *Ppar-γ* and its co-receptor and *Ppar-γ* agonists have already been approved for the treatment of Type 2 diabetes, the authors tested the effect of rosiglitazone on malaria infection. Indeed, rosiglitazone protected mice from ECM and reduced parasite levels in *P. chabaudi chabaudi* infections in a CD36 dependent manner [31]. The same authors investigated the effect of rosiglitazone on *pf-*glycosylphosphatidylinositol induced TLR2 signaling through JNK, ERK1/2 and p38 leading to the degradation of IκBα, a precursor of NF-κB. Rosiglitazone inhibited phosphorylation of JNK, ERK1/2 and p38 and IκBα degradation in a dose dependant manner [31]. In a phase I/II trial rosiglitazone was given to patients with uncomplicated malaria and increased parasite clearance and a reduced inflammatory response to the infection were observed [46].

In addition, different alternative splicing variants of *Ppar-γ* exist and variations in the 5′ UTR affect translational efficiency [47]. Numerous genetic variations in human *Ppar-γ* are present and have been linked to insulin resistance and adipocyte differentiation (reviewed in [48]). Few links have been made between diabetes and malaria so far even though sequestered parasites have been found in adipose tissues of human, monkeys and mice [27], [49], [50], [51]. C57BL/6J-*ob/ob* mice are a model for Type 2 diabetes and obesity. These mice were protected from ECM and survived significantly longer than wild-type mice when infected with *P. berghei* [52]. The same mice lowered blood glucose levels upon injection of inositol phosphoglycans from *P. yoelii* [53]. Although hypoglycaemia rather than hyperglycaemia is associated with malaria, similar genes might play a role in the regulation of blood glucose levels.

*Tsen2*, the gene downstream of *Ppar-γ*, is a tRNA splicing endonuclease, which catalyzes the first step in RNA splicing by removing introns. We observed haplotype specific expression patterns for *Ppar-γ* and *Tsen2* strengthening the fact that the expression of these genes are based on different haplotypes in the UTR and these in turn could play a role in the variable outcome of the malaria infection in various mouse strains. These data together strengthen the importance of *Ppar-γ* in malaria infection and makes it a likely target for the intervention of CM. *Tsen2* might also play a role either in concert with *Ppar-γ* or alone.

Because the probability scores in the HAM analysis are based in part on the number of strains sharing the phenotype, some of the smaller peaks on other chromosomes may prove to be equally important. For example, we found two minor peaks on chromosome 11 with a -Log_10_P score of 4.08 and 3.96 that flank the char8 locus [10]. QTL analyses of crosses between other susceptible and resistant mouse strains provide additional evidence for the importance of multiple genes in *P. berghei* infection [15], [16], [17], [18].

In sum, we have generated a vast amount of phenotypic data for 32 mouse strains infected with malaria. Only a few inbred strains have been characterized before and the available data for these new strains allows scientists to choose the strain best suited for their research. Without conducting expensive crosses, we have mapped survival to a new locus on chromosome 6 by using a combination of HAM and EMMA and our data support the importance of *Ppar-γ* in malaria infection. Future studies will be needed to define the role of *Ppar-γ* and *Tsen2* in infection and association studies in malaria endemic regions might reveal protective or susceptible polymorphisms in human populations.

## Materials and Methods

### Ethic Statement

All animal experiments were approved by the Institutional Animal Care and Use Committee (IACUC) and conducted in agreement with the NIH policy.

### Mice

The 32 inbred strains used were purchased from The Jackson Laboratories (JAX): 129S1/SvlmJ, AKR/J, BALB/cByJ, BALB/cByJ, BTBRT+tf/J, BUB/BnJ, C3H/HeJ, C57BL/6J, C57BR/cdJ, C58/J, CBA/J, CE/J, DBA/2J, FVB/NJ, I/LnJ, KK/HIJ, LG/J, MA/MyJ, MRL/MpJ, NOD/LtJ, NON/LtJ, NZO/HILtJ, NZW/LacJ, P/J, PL/J, RIIIS/J, SJL/J, SM/J, SWR/J and the three wild-derived inbred strains: WSB/EiJ, PERA/EiJ, CZECHII/EiJ. Eight to twelve week old mice were used in the study. Mice were housed in a pathogen free facility at the Genomics Institute of the Novartis Research Foundation (GNF) and all experiments were approved by the Institutional Animal Care and Use Committee (IACUC) and conducted in agreement with the NIH policy.

### Infection and parasites

The *Plasmodium berghei* ANKA strain *PbGFP–LUC_SCH_* [54] was used for all infections. This strain expresses a GFP-luciferase fusion protein under the schizont specific promoter of the *P. berghei ama1* gene. The fusion protein is expressed in the schizont stage but also in the very early ring stage. Parasites from frozen stocks of this strain were propagated and maintained in BALB/cByJ mice. Four female and four male mice of each strain were infected intraperitoneally with 1×10^6^-parasitized red blood cells obtained from a donor mouse. The parasites were preserved in Alsever's solution containing 10% glycerol and stored in liquid nitrogen.

### Measurement of body temperature

We used Bio Medic Data Systems, Inc. (BMDS) implantable microchip system, specifically the DAS-5002 Notebook System and the IPTT-300 Implantable Programmable Temperature Transponders, to identify and record body temperature from each mouse. The DAS-5002 Notebook System is a portable hand-held reader-programmer used to program and scan BMDS temperature microchips. Thus, body temperature can be collected from each animal with the use of the BMDS hand-held wand while the animal is in its own cage without any need for restraint. The chips are placed under the skin with a 16-gauge needle placement device. Rodents may be lightly anesthetized with isoflurane to minimize any undue movement during the chip implantation procedure.

### Visualization and quantification of luciferase activity in whole bodies and dissected organs of infected animals

Luciferase activity in mice were visualized through the imaging of whole bodies or dissected organs by using an intensified- charge-coupled device (I-CCD) photon counting video camera of the *in vivo* Imaging system (IVIS 100, Xenogen). The animals were anesthetized by isoflurane and injected intraperitoneally with D-luciferin dissolved in PBS (100 mg/kg of body weight). Animals were kept anesthetized and measurements were done between 2 to 4 min after injection with D-luciferin. Bioluminescence imaging was acquired with a 15 cm FOV (field of view), a medium binning factor and exposure times of 10–60 s. Individual organs were obtained by dissection of animals without perfusion and placed in a Petri dish and imaged with a 10 cm FOV, a medium binning factor, and exposure times between 10–60 s. Measurements were done using fixed time and region-of-interest settings with the programs LIVING IMAGE (Xenogen) and IGOR-PRO (Wavemetrics). Relative photon counts and intact organs were related to the total photon counts of all the organs.

### Haplotype Associated Mapping (HAM) and efficient mixed-model association (EMMA)

The detailed algorithm underlying the Haplotype Association Mapping (HAM) method [23], [24] and the efficient mixed-model for association (EMMA) mapping model [25] have been previously described

Briefly, the SNPster software forms haplotypes from three-SNP windows based on SNP data available for the mouse diversity panel, MDP [55]. These inferred haplotypes are used as factors to calculate F statistics by ANOVA with various input traits. In this study average survival was used as trait.

The EMMA algorithm is based on the mixed-model where the pair-wise relatedness between all individuals is estimated and then fitted to the vector of the phenotype thereby decreasing false positive and false negatives. EMMA is available as an R package implementation (http://mouse.cs.ucla.edu/emma/index.html).

### Statistical analysis

One-way analysis of variance (ANOVA) followed by Tukey-Kramer posttest (alpha = 0.05) and two-tailed Pearson correlations were performed in GraphPad Prism. T test and hyper-geometric probability distributions were calculated in Excel. The proportion of variance explained by the locus was calculated in R by fitting the phenotypes against the genotypes to a linear model.

## Supporting Information

Figure S1 Visualization of luciferase expression in living mice and dissected organs. Mice were infected with *Plasmodium berghei* ANKA strain PbGFP-LUC_SCH_ that expresses a GFP-luciferase fusion protein under the schizont specific promoter of the *P. berghei ama1* gene. Anesthetized mice were injected with D-luciferin and kept under anesthesia during the measurement. Luciferase activity in mice as visualized by using an I-CCD video camera. Rainbow images show the relative level of luciferase activity ranging from low (blue), to medium (green), to high (yellow, red). Note that the time of exposure and the total photon counts are different between different days. Whole body images of DBA/2J females throughout the infection starting on day 3 (A), to day 10 (B), to day 14 (C) where the mice were euthanized and the organs dissected and measured (D).
(8.58 MB TIF)

Table S1 Summary of phenotypes and statistics. The mouse strains were grouped according to their ancestry [62]. The average and standard deviation for survival, terminal parasitemia and terminal body temperature are indicated for all mice of a strain combined as well as divided by sex. The number of mice (N) analyzed per experiment is indicated. Strains that showed statistically significant differences by un-paired students t-test are colored in red. The relative luciferase expression in various organs (brain, spleen, lung and liver) and the total luciferase expression of the sum of organs are shown for each strain but not divided by sex due to the small numbers of mice analyzed by strain. Correlations for the various mouse strains between different traits were calculated in Prism and correlations in green are statistically significant.
(0.04 MB XLS)

Table S2 Matrix for ANOVA analysis for Figure 1 and Figure 2 C. A) One-way ANOVA analyses were calculated for survival, total luciferase counts and terminal body temperature for each strain compared to another. B) One-way ANOVA analyses were calculated for luciferase expression in spleen, liver lungs and brain of all mouse strains. Statistical significant differences between strains are indicated.
(0.03 MB XLS)
