# Supplementary figures and images for: Genome Wide Analysis of Inbred Mouse Lines Identifies a Locus Containing Ppar-γ as Contributing to Enhanced Malaria Survival
Source: PLoS One. 2010 May 28;5(5):e10903. doi: 10.1371/journal.pone.0010903 (PMC2878346; doi:10.1371/journal.pone.0010903)

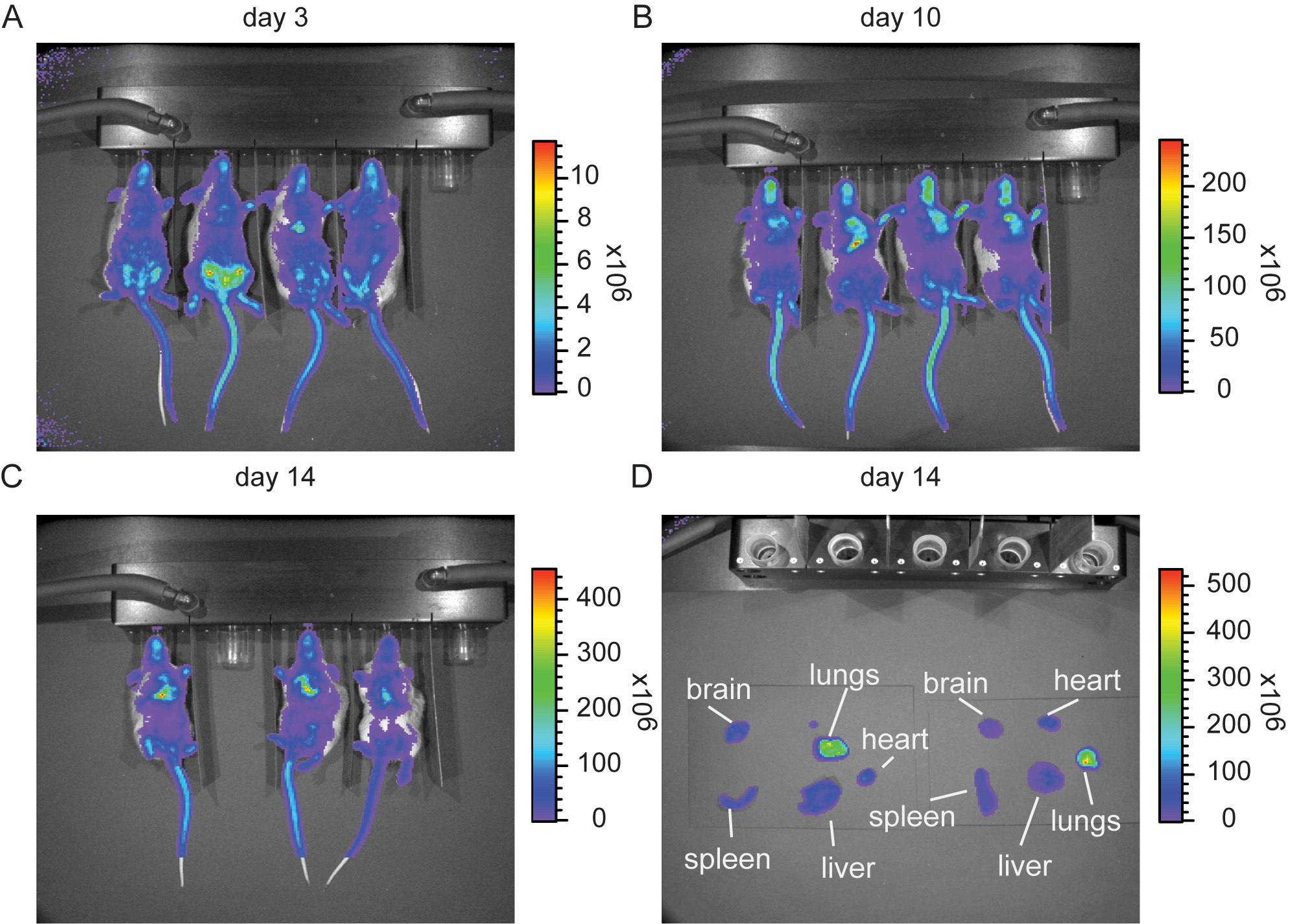

Supplement: Figure S1 — Visualization of luciferase expression in living mice and dissected organs. Mice were infected with Plasmodium berghei ANKA strain PbGFP-LUCSCH that expresses a GFP-luciferase fusion protein under the schizont specific promoter of the P. berghei ama1 gene. Anesthetized mice were injected with D-luciferin and kept under anesthesia during the measurement. Luciferase activity in mice as visualized by using an I-CCD video camera. Rainbow images show the relative level of luciferase activity ranging from low (blue), to medium (green), to high (yellow, red). Note that the time of exposure and the total photon counts are different between different days. Whole body images of DBA/2J females throughout the infection starting on day 3 (A), to day 10 (B), to day 14 (C) where the mice were euthanized and the organs dissected and measured (D). (8.58 MB TIF) [file pone.0010903.s001.tif]
